# Automated Quantification and Integrative Analysis of 2D and 3D Mitochondrial Shape and Network Properties

**DOI:** 10.1371/journal.pone.0101365

**Published:** 2014-07-02

**Authors:** Julie Nikolaisen, Linn I. H. Nilsson, Ina K. N. Pettersen, Peter H. G. M. Willems, James B. Lorens, Werner J. H. Koopman, Karl J. Tronstad

**Affiliations:** 1 Department of Biomedicine, University of Bergen, Bergen, Norway; 2 Department of Biochemistry (286), Nijmegen Centre for Molecular Life Sciences, Radboud University Medical Centre, Nijmegen, The Netherlands; Tufts University, United States of America

## Abstract

Mitochondrial morphology and function are coupled in healthy cells, during pathological conditions and (adaptation to) endogenous and exogenous stress. In this sense mitochondrial shape can range from small globular compartments to complex filamentous networks, even within the same cell. Understanding how mitochondrial morphological changes (*i.e.* “mitochondrial dynamics”) are linked to cellular (patho) physiology is currently the subject of intense study and requires detailed quantitative information. During the last decade, various computational approaches have been developed for automated 2-dimensional (2D) analysis of mitochondrial morphology and number in microscopy images. Although these strategies are well suited for analysis of adhering cells with a flat morphology they are not applicable for thicker cells, which require a three-dimensional (3D) image acquisition and analysis procedure. Here we developed and validated an automated image analysis algorithm allowing simultaneous 3D quantification of mitochondrial morphology and network properties in human endothelial cells (HUVECs). Cells expressing a mitochondria-targeted green fluorescence protein (mitoGFP) were visualized by 3D confocal microscopy and mitochondrial morphology was quantified using both the established 2D method and the new 3D strategy. We demonstrate that both analyses can be used to characterize and discriminate between various mitochondrial morphologies and network properties. However, the results from 2D and 3D analysis were not equivalent when filamentous mitochondria in normal HUVECs were compared with circular/spherical mitochondria in metabolically stressed HUVECs treated with rotenone (ROT). 2D quantification suggested that metabolic stress induced mitochondrial fragmentation and loss of biomass. In contrast, 3D analysis revealed that the mitochondrial network structure was dissolved without affecting the amount and size of the organelles. Thus, our results demonstrate that 3D imaging and quantification are crucial for proper understanding of mitochondrial shape and topology in non-flat cells. In summary, we here present an integrative method for unbiased 3D quantification of mitochondrial shape and network properties in mammalian cells.

## Introduction

Mitochondria play diverse roles in eukaryotic cell physiology in that they serve as producers of ATP and constitute essential hubs of metabolism and signal transduction. The organelle is composed of a mitochondrial outer membrane (MOM) that surrounds an inner membrane (MIM), which is highly folded and encloses the matrix compartment where metabolic enzymes and the mitochondrial genome reside. Mitochondria operate as main tranducers of cellular energy and house enzyme systems for β-oxidation, the TCA cycle, ketogenesis, and oxidative phosphorylation (OXPHOS). The OXPHOS machinery is embedded in the MIM, and consists of the electron transport complexes and the ATP synthase. In this system, electron flow drives transmembrane transport of protons, which generates the proton gradient utilized for ATP production by ATP synthase [1]. Although mitochondria are characterized by having some degree of genetic and metabolic autonomy their function is intricately linked to that of the cell. In this sense, evidence has been provided that bidirectional mitochondria-cell communication through major signaling pathways occurs in cellular homeostasis, growth, survival and death. Hence, exogenous and endogenous factors including nutritional status, pharmacological modulation, cytosolic signal transduction and the presence of pathological mutations may (in) directly affect mitochondrial function [2]–[7].

In living cells, mitochondria can form a large tubular assembly (“a reticulum”) extending throughout the cytosol, which is often close to other cellular compartments like the nucleus, endoplasmatic reticulum (ER) and cytoskeleton [8]–[10]. The cellular volume fraction occupied by mitochondria varies between cell types and with metabolic condition [11], [12]. Mitochondrial morphology is very dynamic and can shift between fragmented structures and filamentous networks, via mitochondrial fission and fusion events [13]. Mitochondrial morphology is directly controlled by the balanced action of fission and fusion proteins including the optic atrophy 1 (OPA1) protein, mitofusins (Mfn1 and 2), dynamin-related protein 1 (Drp1) and the fission 1 (Fis1) protein [6], [14]–[16].

Impairments in the regulation and function of mitochondria may severely affect cellular homeostasis, and such defects have been associated with aging and disease, including metabolic disorders, cancer and neurodegeneration [17], [18]. For example, mitochondrial morphological aberrations have been observed in muscle and skin cells of patients with inherited mitochondrial disease [19], [20]. Moreover, chronic (72 h) inhibition of the first OXPHOS complex (complex I or CI) by rotenone (ROT), stimulated mitochondrial filamentation (*i.e.* length and degree of branching) in primary human fibroblasts [21]. In endothelial cells, bioenergetic stress induced by OXPHOS inhibitors triggered specific changes in mitochondrial morphology, possibly indicative of the cellular stress level and thereby cell survival [22]. This suggests that mitochondrial dynamics and spatial localization are linked to mitochondrial and cellular (dys) function [5], [6], [14], [16], [21], [23], [24].

A proper understanding of the relationships between mitochondrial morphology and physiology demands automated quantitative methods to analyze mitochondrial shape. Mitochondrial shape parameters can be obtained using fluorescent cations that specifically accumulate within mitochondria (*e.g.* TMRE, TMRM, rhodamine 123, JC-1) or by genetically introducing mitochondria-targeted fluorescent proteins [25], [26]. At present, mitochondrial shape analysis is primarily performed by automated computer-assisted analysis of 2-dimensional (2D) fluorescence microscopy images, employing suitable types of software and (custom) algorithms [6], [27]. These strategies work best on cultured cells with a relatively flat morphology. For cells displaying a substantial “thickness” in the axial (z) direction, three-dimensional (3D) image acquisition and analysis are required. In principle, confocal microscopy allows acquisition of axial image sequences (“z-stacks”) for 3D quantification. However, relatively few strategies optimized for mitochondrial analysis have been reported [6]. One such strategy involves creation of a semi-3D image by collapsing multiple image-sections from a wide-field or confocal z-stack into a single 2D projection [28], [29]. A more advanced method is to generate a 3D representation of the mitochondria from the z-stack and performing a 3D analysis of the mitochondrial objects. Although technically more demanding than 2D procedures, attempts to analyze mitochondria in 3D appeared promising in studies of mitochondrial shape and network properties [6], [22], [25], [30]–[32]. However, to the best of our knowledge, none of the published 3D strategies involves combined and integrative assessment of mitochondrial shape and network properties. In this study, we established and validated such analysis in z-stacks of human endothelial cells (HUVECs) expressing a mitochondria-targeted green fluorescence protein (mitoGFP). The performance of this 3D approach was compared with our previously described 2D algorithm [6], [20], [24], [27], [33]–[40] in healthy cells and during ROT-induced metabolic stress conditions. Our results demonstrate that 3D imaging and quantification are of crucial importance for proper analysis of mitochondrial dynamics in cells displaying a non-flat morphology. Hopefully, the strategy provided in this study may contribute to clarify new relationships between mitochondrial morphology and physiology.

## Materials and Methods

### Cell culture

Phoenix A retroviral packaging cells were used for virus production [41]. The cells were maintained in DMEM with 4.5 g/l glucose (Gibco) supplemented with 10% fetal bovine serum (FBS), 2 mM L-glutamine, 100 U/ml penicillin and 100 µg/ml streptomycin (all from Sigma-Aldrich). Human Umbilical Vein Endothelial Cells (HUVECs) were purchased from Lonza, Basel, Switzerland (C2517A). The cells were cultured in EGM-2 (Lonza), and kept at 37°C and 5% CO_2_. The growth medium was changed every second or third day and the cells were passaged prior to reaching confluence. The maximum passage number used for experiments was 8.

### Cloning and retroviral transduction

HUVECs stably expressing mitochondrial targeted GFP (mitoGFP) were produced by retroviral transduction [41]. Generation of a retroviral vector carrying mitoGFP was initiated by exchanging EYFP in the commercial available pEYFP-mito vector (BD Biosciences, Clontech) with EGFP. GFP from pEGFP-N1 and YFP from pEYFP-mito were excised using the restriction enzymes BamHI and BsrGI. The products were separated by gel electrophoresis and the pEGFP insert and the vector with the mitotargeting sequence were gel-purified using GFX columns (GE Healthcare). EGFP was then cloned into the vector fragment, generating the pEGFP-mito vector. Further, pEGFP-mito was cut with NheI before converting overhangs to blunt ends followed by further cutting with NotI. The resultant fragment was cloned into the retroviral vector pCGFP [42] previously cut with BamHI, blunt-ended, and cut again with NotI to remove the existing GFP fragment. The fragments were separated by gel electrophoresis and gel-purified using GFX columns prior to ligation. This resulted in generation of the pCEGFP-mito vector. Correct orientation of the inserts was controlled using restriction enzymes XbaI and NotI, and plasmids with correct promoter orientation were sequenced. All enzymes and buffers used during cloning procedures were from New England Biolabs. Phoenix A packaging cells were transfected by CaCl_2_ precipitation in presence of chloroquine (Sigma-Aldrich) with the pCEGFP-mito retroviral vector. At 6 h post-transfection, the medium was replaced with fresh DMEM containing 10% FBS and cells were grown for 12 hours. The medium was then replaced with EGM-2 medium and the cells were grown for additionally 24 hours to produce retroviruses. Conditioned media was collected, filtered through 0.45 µm-pore-size polysulfonic filters and added to the HUVEC culture together with protamine sulfate (5 µg/ml) (Sigma-Aldrich). Fresh EGM-2 medium was again added to the Phoenix A packaging cells, and after additional 24 hours virus were harvested and added to the HUVECs for a second round with virus infection. After the first overnight incubation with virus-containing medium, the cells were incubated in fresh EGM-2 for 8 hours, prior to an additional overnight incubation in the presence of the virus vector. After infection, HUVECs were cultured before GFP-positive cells were sorted on a FACSAria Cell Sorter (BD Biosciences).

### Confocal microscopy

12,000 HUVECs were seeded onto 18 mm coverslips and were allowed to adhere under routine culturing conditions for 2–4 hours before treatments were added. The cells were then incubated for 72 hours before they were fixed with cold (4°C) 3.7% paraformaldehyde at room temperature for 30 minutes. The coverslips were rinsed in PBS and water before they were dried and mounted onto a cover glass using Vectashield mounting medium with DAPI (Vector Laboratories, California, US). The z-stack images of mitoGFP were acquired on a Zeiss LSM 510 Meta confocal microscope (Carl Zeiss, Oberkochen, Germany) using a Plan-Apochromat 63×1.40 NA oil objective. Excitation wavelength was 488 nm and emission was detected using a long pass filter from 505 nm. Image pixel size was 0.0845 µm (x and y) and bit depth 8, z-step size 0.364 µm, and pinhole diameter 96 µm.

### Image processing and analysis

All image processing and analysis were performed using the Image-Pro Plus software (version 7.0) with the SharpStack Total deconvolution and 3D Constructor modules (Media Cybernetics, Inc., Washington, USA). The blind deconvolution algorithm of this software is the same found in the AutoQuant software (Media Cybernetics, personal communication). The datasets were first calibrated using the acquisition system parameters, and cropped to exclude unnecessary regions, before processing by 3D blind deconvolution. For comparative purposes, the images were processed using spatial filtering and analyzed as described in [27]. For the Fast Fourier Transform (FFT) processing, a circular area of interest (AOI; radius setting 2, 5 or 10, as specified) was defined in the center of the spectrum in the frequency domain. Projections of multiple z-stack sections into one image were performed either by averaging or by generating a maximum intensity composite (MIC). For 3D analysis, the z-stacks were loaded into the 3D Constructor module using no sub-sampling. A 3D iso-surface was created without further filtering or simplification prior to volumetric shape measurements. Regions of interest (ROIs) were selected stochastically in mitochondria-rich parts of the cytoplasm. With respect to mitochondrial network analysis, the objects were skeletonized using standard processing operations (medial axis transform), which involved an intensity threshold, followed by thinning and then pruning of the objects. The threshold intensity corresponded with the respective threshold used in the parallel shape analysis, as indicated in the individual experiments. We used 2 thinning iterations to create the topological skeleton, and 2 pruning iterations to remove small extensions due to irregularities in the objects (i.e. noise such as single-pixel bumps) but not significant parts of the structures. The resulting mitochondrial skeleton was vectorized to identify and count/measure branches (skeletal backbone), end points and branch points as graphic vectors/points. This was also used to measure distance map values (i.e. how far from the edge of the object any pixel/voxel lies) in order to determine branch diameter and volume. All these image operations are conventional and can be applied in suitable image processing software. In Image-Pro Plus, these operations have been integrated in a consecutive manner in the built-in “NeuronAnalyzing” macro, which we employed in this study.

## Results

### Analysis of the shape of synthetic objects in 2D and 3D

**Figure 1:**
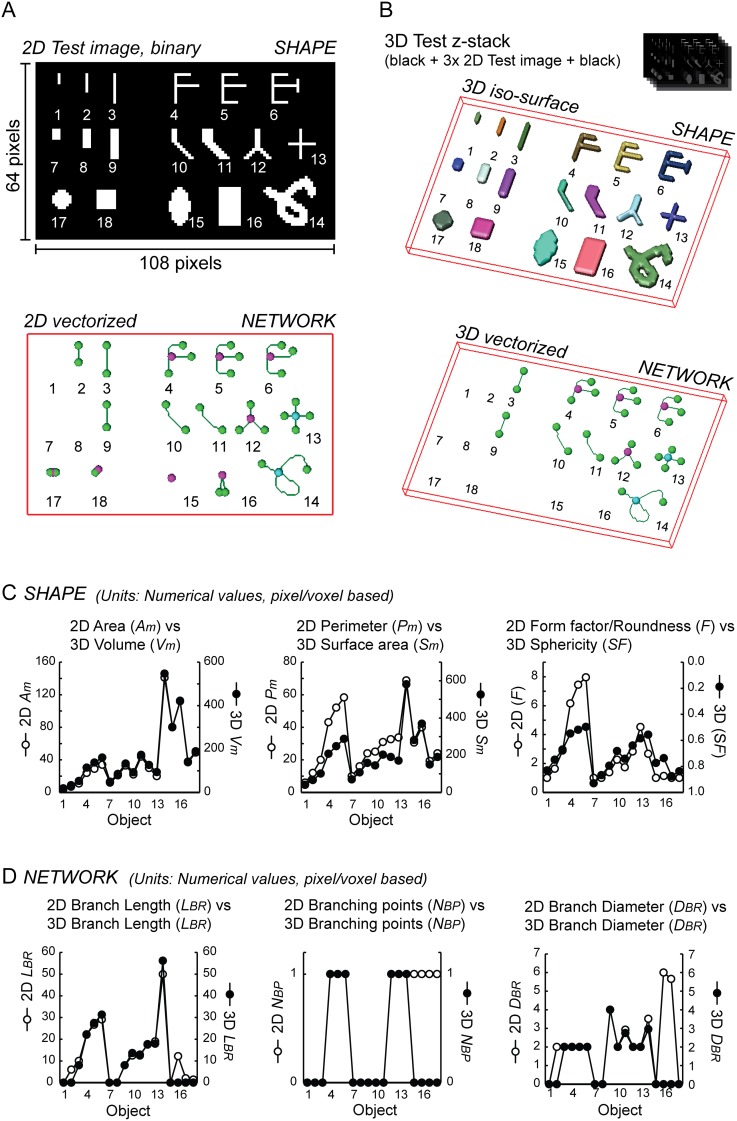
Shape and network analysis of synthetic objects in 2D and 3D. (**A**) The image (upper panel) shows the binary (black and white) 2D test image with synthetic objects. The lower panel shows the vectorized skeletons produced as part of the network analysis. Object shape and network properties were analyzed in two separate operations. (**B**) A 3D test z-stack was made by combining three copies of the 2D test image, flanked by an empty (black) image on the top and bottom. The upper panel shows 3D volumetric models made by iso-surface rendering (voxel size *x = y = z* = 1), and the lower panel display the 3D vectorized skeletons. Shape (**C**) and network (**D**) data of descriptors with a correspondent meaning in 2D and 3D are shown. The descriptor variables are further explained in **Table 1**.

Our first objectives were to determine: (**i**) if the previously described principles of 2D analysis [21], [27] could be translated into 3D analysis, and (**ii**) if mitochondrial filament properties could be quantified analogous to neuronal networks (see Materials and Methods). To allow proper interpretation of the parameters describing mitochondrial shape and network properties (“descriptors”) a 2D test image was used. This image contained mitochondria-like synthetic objects of relevant size (**Fig. 1A**). A 5-section z-stack was generated by layering of 3 copies of the 2D image between an empty (black) image on the top and bottom of the series (**Fig. 1B**). For the 2D image, descriptors of mitochondrial shape were quantified as previously described in detail [27]. For analysis of the 3D image, an iso-surface 3D (volumetric) model was generated from the z-stack. In addition to the shape analysis, the network analysis algorithm was used to skeletonize objects (in 2D and 3D), to perform vectorization, and to identify and quantify branch properties, branching points and endpoints. To allow faithful quantitative comparison of objects in the 2D and 3D test images, we selected descriptors with a correspondent meaning in 2D and 3D (**Table 1**). Plotting the numerical value for each shape descriptor and each object in the 2D and 3D image gave similar results (**Fig. 1C and D**). In this sense, the area of each 2D object (*A_m_*) was virtually identical with the volume (*V_m_)* of the corresponding 3D object (**Fig. 1C**; left panel). The 2D object perimeter (*P_m_*) and 3D object surface area (*S_m_*) also gave similar profiles (**Fig. 1C**; middle panel). Of note, an increase in length and degree of branching of the test objects induced a greater increase in *P_m_* than in *S_m_*. To assess the “complexity” of 2D objects we analyzed the 2D formfactor (*F*), which is a measure of mitochondrial length and degree of branching [27], [33]. In the 3D image, the sphericity factor (*SF*) was used as an equivalent measure of object complexity (**Fig. 1C**; right panel). *F* and *SF* gave similar profiles when plotted with inverse scaling in the same diagram (**Fig. 1C**; right panel). This is expected since there is an inverse relationship between *F* and *SF* as descriptors of circular (2D)/spherical (3D) object properties (**Table 1**). Taken together, the above results demonstrate that the shape/volume descriptors can be used to discriminate between objects of different width, objects with simple *vs.* complex morphologies, and circular (2D) *vs.* spherical (3D) objects.

**Table 1:** Parameters of mitochondrial morphology.

Analysis modality	Descriptor		Comment
**2D Shape analysis**	Count	*N_ROI_*	Number of mitochondria in the ROI.
	Area	*A_m_*	Area of mitochondrion (per object).
		*A_m,ROI_*	Total mitochondrial area in the ROI.
	Perimeter	*P_m_*	Length of the mitochondrial outline (per object).
		*P_m,ROI_*	Total length of the mitochondrial outline in the ROI.
	Formfactor (Roundness)	*F*	Calculated as (P_m_^2^)/(4·π·A_m_). Circular objects will have an *F*-value close to 1, other shapes will have *F*>1.
**3D Shape analysis**	Count	*N_ROI_*	Number of mitochondria in the ROI.
	Volume	*V_m_*	Volume of mitochondrion (per object).
		*V_m,ROI_*	Total mitochondrial volume in the ROI.
	Surface area	*S_m_*	Surface area of mitochondrion (per object).
		*S_m,ROI_*	Total mitochondrial surface area in the ROI.
	Sphericity factor	*SF*	Calculated as *SF* = (6·*V_m_*)/(*D_m_*·*S_m_*), where *D_m_* is the equivalent diameter. For a spherical object *SF* is close to 1, all other shapes have an *SF*<1.
**2/3D Network analysis**	Branch count	*N_BR_*	Number mitochondrial branches, i.e. detached filaments and filaments attached to branch points.
	Branch points	*N_BP_*	Number of points wherein three or more mitochondrial branches are attached.
	Branch length	*L_BR_*	Length of the mitochondrial branch when unfolded to its maximal length (per object).
		*L_BR,ROI_*	Total length of the mitochondrial branches in the ROI.
	Branch diameter	*D_BR_*	Mean diameter of the mitochondrial branch.
	Branch volume	*V_BR_*	Volume of the mitochondrial branch (per object). Calculated as V_BR_ = (π·D_BR_^2^/4) ·L_BR._
		*V_BR,ROI_*	Total volume of mitochondrial branches in the ROI.
	End points	*N_EP_*	Number of branch endpoints (all objects)
**2/3D Network - Shape**	Branch (filament) length per mitochondrion		*L_BR,ROI_/N_ROI_*
**Integrative analysis**	Branch (filament) length per biomass		*L_BR,ROI_/A_m,ROI_ (2D); L_BR,ROI_/V_m,ROI_ (3D)*
	Branch number per mitochondrion		*N_BR_/N_ROI_*
	Branch number per biomass		*N_BR_/A_m,ROI_ (2D); N_BR_/V_m,ROI_ (3D)*
	Branching point frequency - branch length		*N_BP_/L_BR,ROI_*
	Branching point frequency - biomass		*N_BP_/A_m,ROI_ (2D); N_BP_/V_m,ROI_ (3D)*

**Legend:** ROI, region of interest.

### Analysis of the network properties of synthetic objects in 2D and 3D

To obtain topological information, network analysis was performed on both the 2D and 3D test datasets (**Fig. 1D** & **Table 1**). In case of filamentous objects the profiles of branch length (*L_BR_*), number of branching points (*N_BP_*) and branch diameter (*D_BR_*) were similar in the 2D and 3D case. Numerical data for spherical/non-filamentous objects were only generated by the 2D analysis protocol but not by the 3D analysis algorithm. The latter was caused by the property of the 2D network rendering procedure to generate small and branched structures that did not properly reflect the original objects. Although unrepresentative structures can be easily removed from the analysis using an object filtering strategy (*e.g.* by discarding objects with a short branch length), they were included here for comparative purposes. We observed that the *L_BR_* and *D_BR_* descriptors allowed a logical discrimination between objects of different filament length and diameter, respectively (**Fig. 1D**; left and right panels). Quantification of the *N_BP_* parameter yielded similar results for 2D and 3D objects (**Fig. 1D**; middle panel).

### Blind deconvolution improves the quality of quantitative mitochondrial analysis

**Figure 2:**
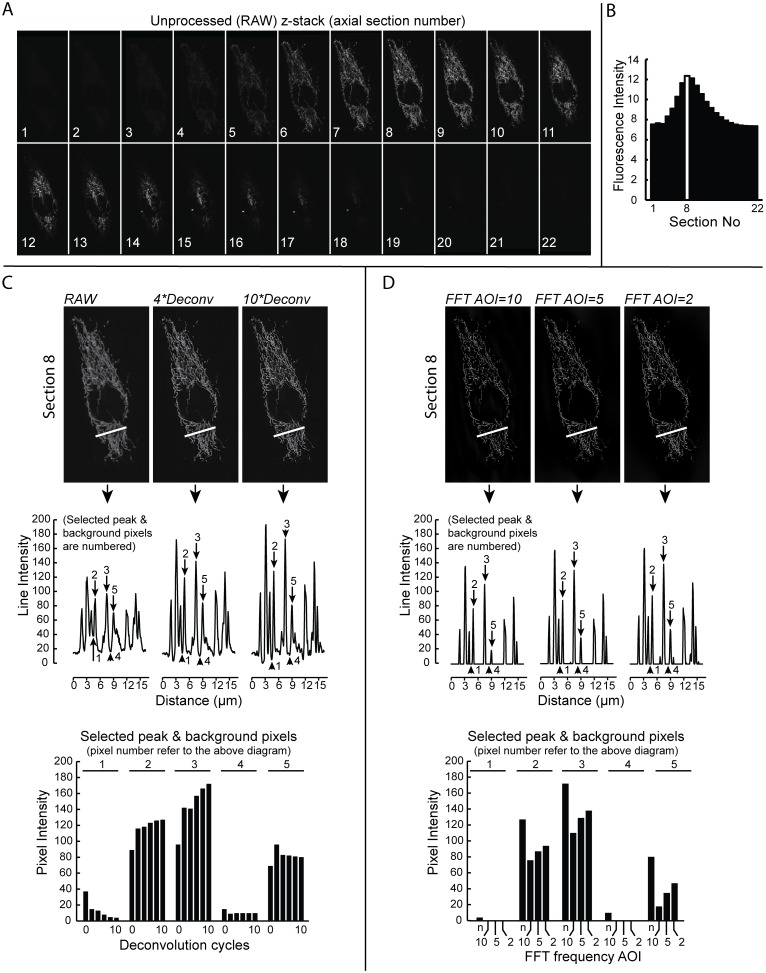
Image optimization for mitochondrial segmentation in 3D z-stacks. (**A**) The images show all sections of an unprocessed (“RAW”) z-stack of a HUVECexpressing mitoGFP. (**B**) The histogram shows the cumulative pixel fluorescence intensity in the individual z-stack sections (RAW). The highest intensity column (section 8) is highlighted in white. (**C**) The effect of 3D blind deconvolution (“Deconv”) on the S/N ratio. Fluorescence intensity was measured across the indicated line (upper panel) before and after 2, 4, 6, 8 or 10 deconvolution cycles. The figure shows z-stack section 8 (upper panel) and the associated line intensity diagrams (middle panel) derived after 0 (“RAW”), 4 (“4*Deconv”) and 10 (“10*Deconv”) deconvolution cycles. The line intensity peaks and valleys identify mitochondrial objects/filaments and background, respectively. The intensities of five selected peak and background pixels (numbered 1–5, middle panel), before and after 2–10 deconvolution cycles are displayed in the column diagram (bottom panel). (**D**) Usefulness of Fast Fourier Transform (FFT) filtering following deconvolution. The images (upper panel) show z-stack section 8 after FFT filtering using frequency domain area of interest (AOI) radius setting 10, 5 and 2 (including Hi-Pass filtering). The intensity profiles across the same line (see above) are shown (middle panel), and the selected peak/background pixel intensities are plotted in the column diagram (bottom panel) for comparison with the non-FFT filtered image (n, identical to “10*Deconv” in (**C**)).

Confocal microscopy is widely used to acquire 3D image stacks (z-stacks) of cells with fluorescently labeled constituents, including mitochondria. The shape of the fluorescent objects in such images will be blurred due to the point spread function (PSF) of the optics (convolution), leading to difficulties separating nearby mitochondria and their networks. In this sense, “deconvolution” is often applied to remove systemic disturbances including haze, in order to improve image contrast and object segmentation [43]. In this study we performed blind deconvolution with an iterative and constrained algorithm (SharpStack/AutoQuant) which repeats the same computational operations in order to adapt itself to the real PSF of the microscope system. Hence, this method is able to adjust to the specific conditions and specimens, and is designed to perform the best possible deconvolution without exceeding the information available. Due to these capabilities, blind deconvolution (including the algorithm used in this study) has been increasingly employed in cell imaging, and has proven useful in studies of subcellular structures in various contexts [44], [45]. Others have confirmed that this blind deconvolution algorithm maintains the linear relationship in object intensities and their relative intensity changes, which validates the use in quantitative analysis [46]. Here we investigated whether 3D blind deconvolution was suited to optimize mitochondrial shape and network analysis. For this purpose we imaged HUVECs that were retrovirally transduced with a mitochondria-targeted variant of the green fluorescent protein (mitoGFP), by confocal microscopy (**Fig. 2A**). To prevent interference of mitochondrial movement during z-stack acquisition cells were fixed using paraformaldehyde treatment, which preserves well both cellular and mitochondrial structure [32]. The signal-to-noise (S/N) ratio was evaluated using the z-stack section displaying the maximal fluorescence intensity (*i.e.* section 8; **Fig. 2B**). Repeated deconvolution cycles increased the intensity difference between mitochondrial objects and the background, as shown by the intensity plotted across the indicated line profile **(** **Fig. 2C**; top and middle panel). Accordingly, increasing the number of deconvolution cycles reduced the background (non-mitochondrial) fluorescence intensity and increased the peak (mitochondria-specific) signal intensities (**Fig. 2C**; bottom panel with intensities of selected peak and background pixels). Based upon the above analysis we used at least 8 cycles to produce a deconvolved version of the image.

### Fast Fourier transform filtering does not improve mitochondrial analysis

**Figure 3:**
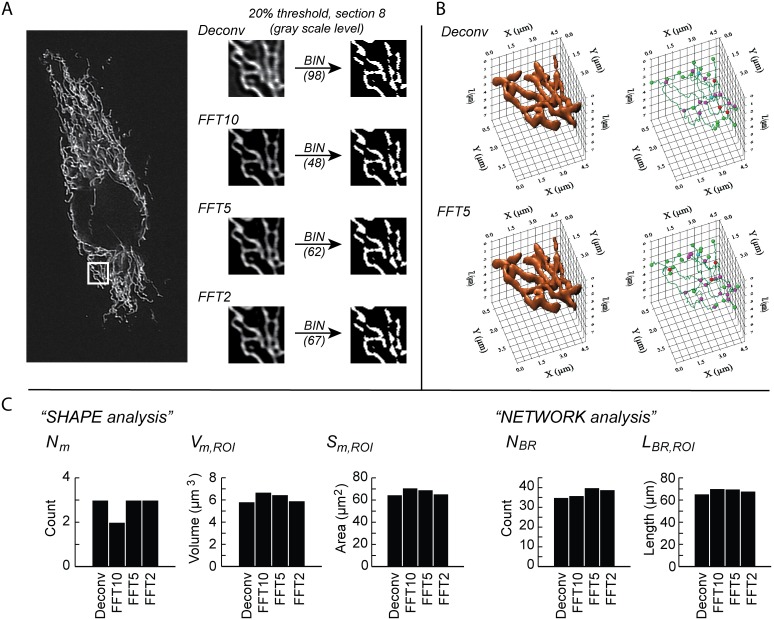
Evaluation of FFT filtering for improving mitochondrial segmentation in z-stacks. A sample z-stack was acquired from a HUVEC expressing mitoGFP (same as in **Fig. 2**). (**A**) The large image shows the highest intensity z-stack section (section 8) after 3D blind deconvolution, and a selected region of interest (ROI) is indicated. The smaller images are magnifications of the ROI before (“Deconv”) and after FFT filtering (including Hi-Pass filtering) with spectrum AOI radius set to 2, 5 or 10, as indicated (*e.g.* “FFT10”). Each FFT filtered ROI-version was binarized (BIN) by selecting the 20% brightest pixels (the corresponding grey tone threshold values are shown in parenthesis). (**B**) 3D volume models of the z-stack ROI were generated and analyzed before and after FFT processing (spectrum AOI radius = 2, 5 or 10). The result after FFT filtering with AOI radius = 5 (“FFT5”) is shown together with the non-FFT processed version (“Deconv”). Shape and network analysis was performed employing the same threshold values as in (A). (**C**) Quantitative data from shape and network analysis in (**B**). Descriptor variables are explained in **Table 1**.

FFT filtering has been employed to optimize fluorescence images for segmentation of mitochondria [28]. Therefore we determined how FFT filtering of the deconvolved z-stack (see above) affected the S/N ratio. Frequency selection in the FFT transformed image (frequency domain) was performed by defining a circular AOI in the center of the spectrum. It was confirmed that FFT filtering highlighted high-intensity (mitochondrial) objects and flattened the non-mitochondrial (background) signal (**Fig. 2D**; top and middle panel). Reducing the AOI radius from 10 to 5 to 2 resulted in a progressive increase in image contrast (**Fig. 2D**; bottom panel). Next, we determined how FFT filtering affected the quantitation of 3D mitochondrial structure by analysis of a region of interest (ROI) within a deconvolved z-stack with and without FFT filtering (**Fig. 3A and B**). The analysis revealed that although FFT filtering apparently increased image contrast, its effects on quantitative mitochondrial shape and network parameters were only minor (**Fig. 3C**). However, careful inspection revealed that FFT filtering somewhat affected the localization of branch points in the mitochondrial network. Given the above results, we decided not to include an FFT filtering step in the image quantification algorithm.

### Extracting 2D mitochondrial shape and network parameters from confocal z-stacks

**Figure 4:**
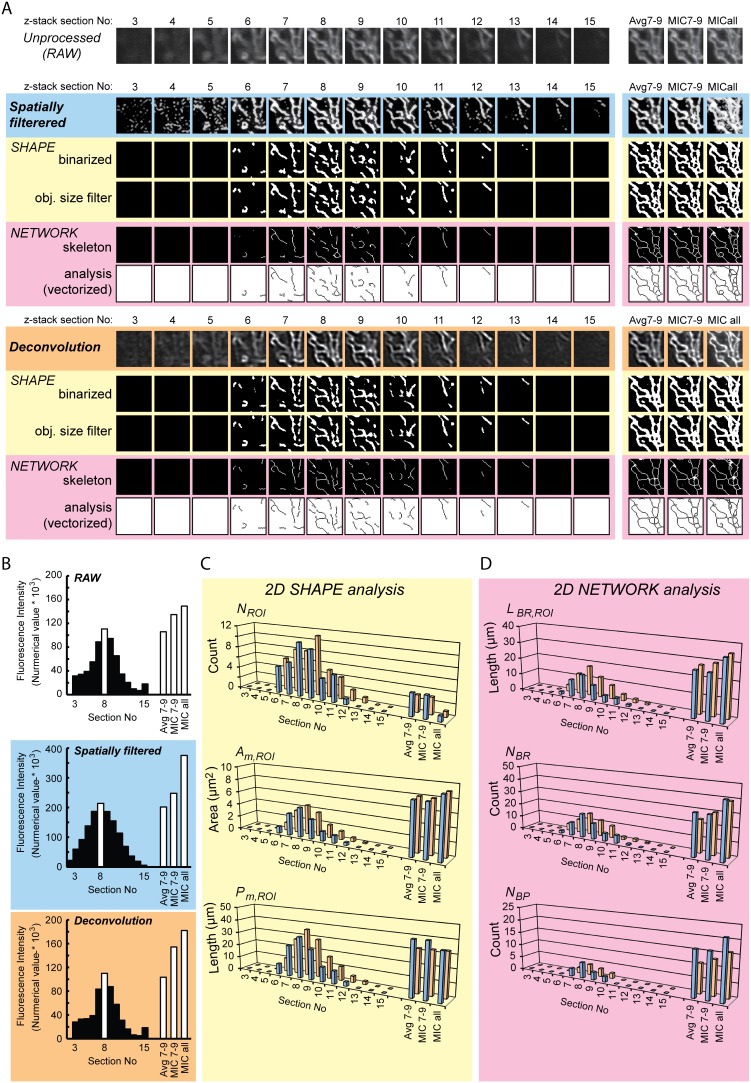
2D and semi-3D analysis of mitochondrial shape and network properties in HUVEC z-stack. A sample z-stack was acquired from a HUVEC expressing mitoGFP, and a region of interest (ROI) was selected (identical to the sample z-stack and the ROI shown in **Fig. 3**). (**A**) The uppermost row shows the ROI sections from the unprocessed z-stack (“RAW”). Further, the ROI was analyzed after spatial filtering (“Spatially filtered”; blue panels), as previously established for 2D mitochondrial analysis [27], or after 3D blind deconvolution as described in the current article (“Deconvolution”; orange panels). 2D projections were made by averaging the three sections of highest intensity (“Avg7–9”), and by creating a maximum intensity composite (MIC) of the three highest intensity sections (“MIC7–9”) or the entire z-stack (“MICall”). These are shown in the right hand panels. Shape analysis (“SHAPE”; yellow panels) was performed after binarization and size filtering. The binarization threshold was fixed to include the 20% brightest pixels in the highest intensity section (section 8). For the 2D projections, the threshold was set to include the 35% brightest pixels in the Avg7–9 and MIC7–9 versions, and 40% for the MICall version. Network analysis (“NETWORK”; pink panels) was performed after skeletonization and vectorization, using the same intensity thresholds as for binarization. (**B**) Section intensity profiles of the unprocessed (“RAW”), spatially filtered and deconvolution processed z-stack ROIs, and the corresponding 2D projections. The resulting quantitative data of mitochondrial shape (**C**) and network (**D**) parameters are shown (see Table 1 for explanations).

Next, we determined whether image deconvolution affected quantification of mitochondrial shape and network properties. For this purpose quantification was carried out on all signal-containing sections in a ROI from a typical z-stack of mitoGFP expressing HUVECs (**Fig. 4A**; “unprocessed (RAW)”). Additionally, representative 2D projections of the z-stack ROI were obtained by: (**i**) averaging of the three sections of highest intensity (**Fig. 4**; “Avg7–9”), (**ii**) generating a MIC image of the three sections of highest intensity (“MIC7–9”) and, (**iii**) calculating a MIC image using all sections (“MICall”). For comparison, all images were processed in two ways using: (**i**) the established spatial filtering 2D technique (**Fig. 4**; “Spatially filtered”; blue panels) developed for flat cells with fluorescently labeled mitochondria [6], [20], [21], [24], [27], [33], [34], [36]–[40], [47], and (**ii**) the deconvolution approach presented in the current paper (**Fig. 4**; “Deconvolution”; orange panels). Following processing, mitochondrial objects were segmented/binarized (see also the next section) and size-filtered (**Fig. 4**; “SHAPE”; yellow panels). Similarly, mitochondrial network properties were analyzed by skeletonization and vectorization (**Fig. 4**; “NETWORK”; pink panels). Relative to the unprocessed situation, the average object pixel intensity in each section was increased by spatial filtering but not by deconvolution (**Fig. 4B**). However, this differential effect on intensity did not significantly influence the quantified shape (**Fig. 4C**) and network properties (**Fig. 4D**). Visual inspection of the average (Avg7–9), MIC and MICall images revealed that these contained a higher amount of mitochondrial pixels, and thus more 2D details, compared to the single highest intensity section within the z-stack (section 8). Mitochondrial objects in these three 2D projections also displayed a higher connectivity as indicated by the relatively low *N_ROI_* value, the relatively high value of *A_m,ROI_*, and the increased values of the network parameters (*L_BR,ROI_*, *N_BR_*, and *N_BP_*). These results confirm (**i**) that deconvolution yields similar results as the established 2D spatial filtering method when analyzing mitochondrial properties in 2D image sections from confocal z-stacks, and (**ii**) that mitochondrial network characteristics are better displayed and analyzed in representative 2D projections of the z-stack, compared to single z-stack sections.

### Influence of threshold settings in mitochondrial 3D analysis

**Figure 5:**
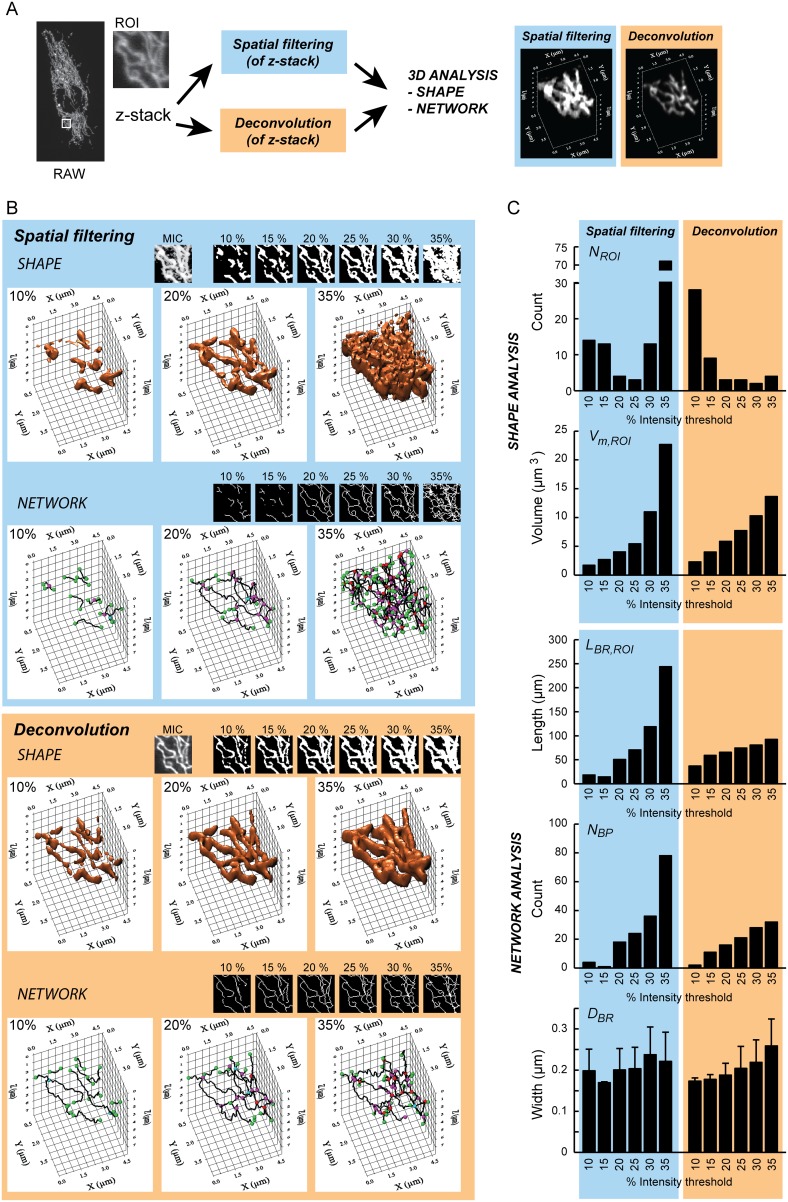
Threshold setting for mitochondrial segmentation in a HUVEC z-stack. A sample z-stack was acquired from a HUVEC expressing mitoGFP, and a region of interest (ROI) was selected (identical to the sample z-stack and the ROI as shown in **Fig. 3**). (**A**) The images show maximum intensity composites (MIC) of the entire unprocessed sample z-stack (“RAW”) and a magnification of the ROI. The figure also displays the comparative strategy to evaluate effects of spatial filtering (“Spatial filtering”; blue panels) and deconvolution (“Deconvolution”; orange panels) for the purpose of 3D mitochondrial segmentation and analysis. (**B**) 3D models of the ROI were generated after spatial filtering, as previously established for 2D mitochondrial analysis [27], and after deconvolution as described in the current paper. The percentages reflect the segmentation thresholds (grey tone values) defining the 10%–35% (as indicated) brightest pixels in the highest intensity section (section 8). The smaller 2D images indicate the effects of threshold setting (MICs created from the processed z-stacks). 3D volume and network models are shown for three of the studied intensity thresholds (10%, 20% and 35%). (**C**) The diagrams show the quantified data from (**B**). Descriptor variables are explained in **Table 1**.

To highlight mitochondrial objects, microscopy images were binarized using an intensity-based threshold operation. Obviously, setting a certain threshold intensity value can influence subsequent 3D analysis of mitochondrial shape and volume properties. The impact of threshold intensity was therefore evaluated on a z-stack with mitoGFP expressing HUVECs that was processed using spatial filtering (see previous section) or by deconvolution and subsequently analyzed (**Fig. 5A**). To allow proper comparison, intensity thresholds were set in such a way that they included a specific fraction (10–35%) of the brightest pixels in the highest intensity section of the z-stack (*i.e.* section 8). Lowering the threshold intensity level (equivalent to including a higher fraction of pixels), reduced the value of the object count parameter (*N_ROI_*), increased object-size parameters (*V_m,ROI_*, *D_BR_*), and increased filament connectivity parameters (*L_BR,ROI_*, *N_BR_*) (**Fig. 5B and C**). It was also observed that threshold adjustments affected mitochondrial segmentation and shape/network parameters less dramatically in the deconvolved z-stack than in the spatially filtered z-stack. For instance, *N_ROI_* remained relatively stable (maximum 2 fold change) in the deconvolved z-stack when using a threshold between 20%–35%, whereas this parameter changed more than 20 fold for the spatially filtered z-stack in the same threshold intensity range. Similarly, *L_BR,ROI_* increased almost 5 fold in the spatially filtered dataset in the 20%–35% threshold range, compared to 1.4 in the deconvolved data. In the spatially filtered z-stack, inclusion of more pixels did not affect *D_BR_*, as after deconvolution; but low thresholds (30% and 35%) introduced a significant amount of noise and thereby an increase in *N_ROI_*. When a 20% threshold setting was applied, similar mitochondrial shape/network data were obtained for the spatially filtered and deconvolved z-stack.

### An automated algorithm for 2D and 3D analysis of mitochondria in HUVECs expressing mitoGFP

**Figure 6:**
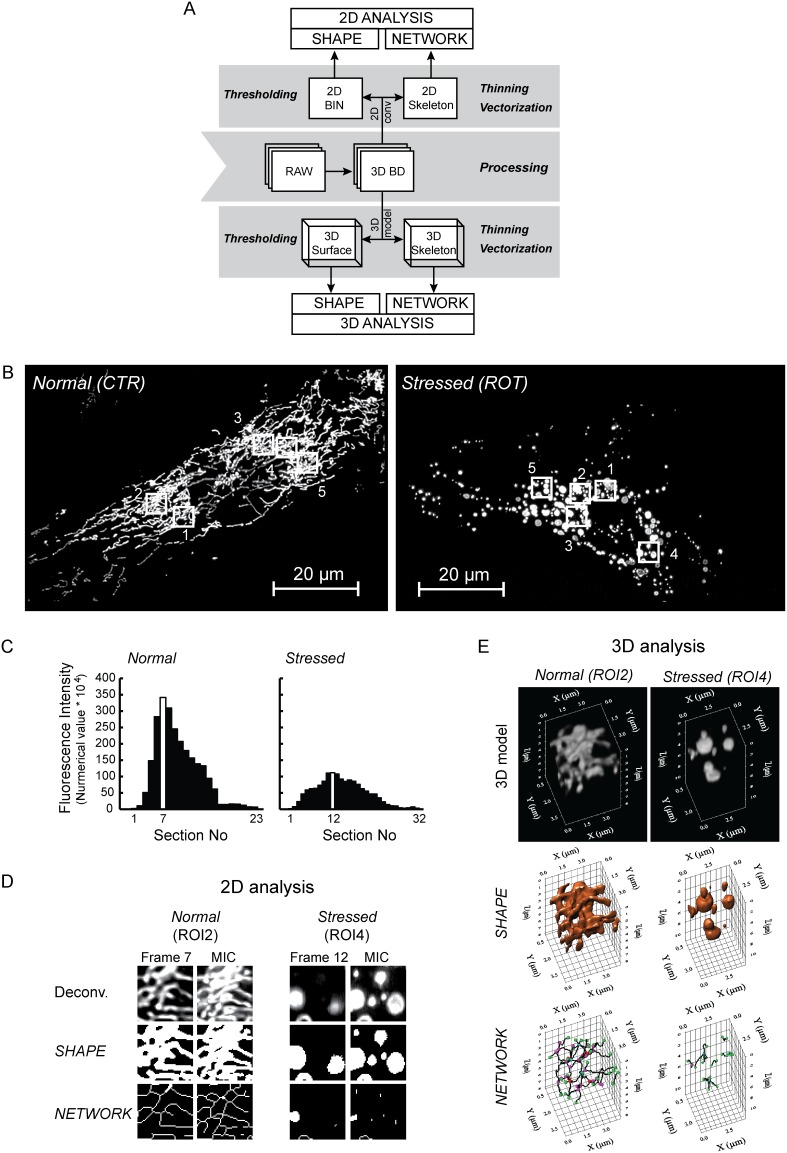
Comparative 2D/3D mitochondrial analysis to detect effects of metabolic stress in HUVECs. Mitochondrial morphology was studied in normal and metabolically stressed (250 nM rotenone, 3 days) HUVECs expressing mitoGFP. (**A**) The figure shows a schematic overview over the procedure established to analyze mitochondrial shape and network properties in 2D and 3D. The boxes represent image outcome, and the arrows indicate mathematical operations. “RAW”, unprocessed z-stack; “3D BD”, z-stack 3D blind deconvolution (10 cycles); “2D BIN”, binarized 2D image (intensity threshold); “2D Network”, skeleton based on the 2D image; “3D Surface”, iso-suface model (intensity threshold); “3D Network”, skeletonized 3D model. The intensity thresholds were determined to give the best reflection of the source image. (**B**) The images are maximum intensity composites (MICs) of the processed z-stacks from an untreated HUVEC (“Normal (CTR)”) and a ROT-treated stressed HUVEC (“Stressed (ROT)”). The z-stacks were processed by deconvolution and a contrast stretch (as described in the current article). Five different regions of interest (ROIs; numbered 1–5 in the images) were selected in each cell. (**C**) The histograms show the intensity profile of the two z-stacks. The highest intensity sections are shown as white columns. (**D**) 2D analysis was performed on the highest intensity frame, and the z-stack MIC. The panels show shape and network representations of one ROI selected from each cell. (**E**) 3D shape and network models were generated and analyzed from each of the ROIs in both cells. Here, the 3D models of one ROI in each cell type are shown.

Based on the results obtained so far, we designed an image processing and analysis algorithm for 2D and 3D analysis of mitochondrial shape and network properties using confocal z-stacks (**Fig. 6A**). The algorithm applies several modules that need to be tuned carefully based on the qualities of the dataset/experiment, and combined to enable an automated sequence of processing and analysis steps. In brief, confocal z-stacks (“RAW”) are processed by 3D blind deconvolution (10 cycles; as described in Materials and methods). Optionally, the contrast may be stretched *e.g.* by employing a minimum intensity threshold to remove low intensity background noise and a maximum intensity limit, before the gray values of the pixels are reassigned to range from 0 to 255 (true for a 8-bit image) (as performed in **Fig. 6** and **Video S1–3**). Further processing and analysis is twofold: **(i)** To enable 2D analysis, the z-stack is projected into a representative 2D MIC image (see previous sections). 2D shape analysis is performed subsequent to binarization of the MIC image, as previously described [27]. For 2D network analysis, the MIC image is skeletonized, vectorized and analyzed using the same threshold level as for the binarization. **(ii)** To enable 3D analysis, a volumetric model of the z-stack is created, and an iso-surface is added to allow mitochondrial shape (3D) measurements. In parallel, 3D network analysis is performed using the same threshold intensity level as for the iso-surface.

### Integrative shape and network analysis of filamentous and non-filamentous HUVEC mitochondria

To test the performance of the method we analyzed mitochondria displaying two characteristic phenotypes, i.e. filamentous *vs.* non-filamentous mitochondria. The z-stacks were acquired from a mitoGFP expressing control HUVEC with filamentous (“Normal”) mitochondria and a metabolically stressed HUVEC (“Stressed”) exposed to the mitochondrial complex I inhibitor rotenone (ROT; (250 nM, 3 days); **Fig. 6B**). The subsequent analysis of 5 perinuclear ROIs (of equal size) from each condition included comparison both of mitochondria with similar (i.e. in the same cell) and non-similar morphology (i.e. normal *vs.* stressed mitochondria), and allowed evaluation of the analytical outcome relative to visual observation. The axial intensity profile revealed that mitochondria were not similarly localized in the two different cells (**Fig. 6C**). A higher number of z-sections were necessary to cover the entire depth of the stressed cell, demonstrating that this cell had rounded up and become “thicker”. 2D analysis was performed on the z-stack section with the highest intensity and the z-stack MIC image (**Fig. 6D**). Data from the MIC image were used for comparison with 3D data. For 3D analysis, volumetric representations were generated for each ROI (*e.g.* **Fig. 6E**).

**Figure 7:**
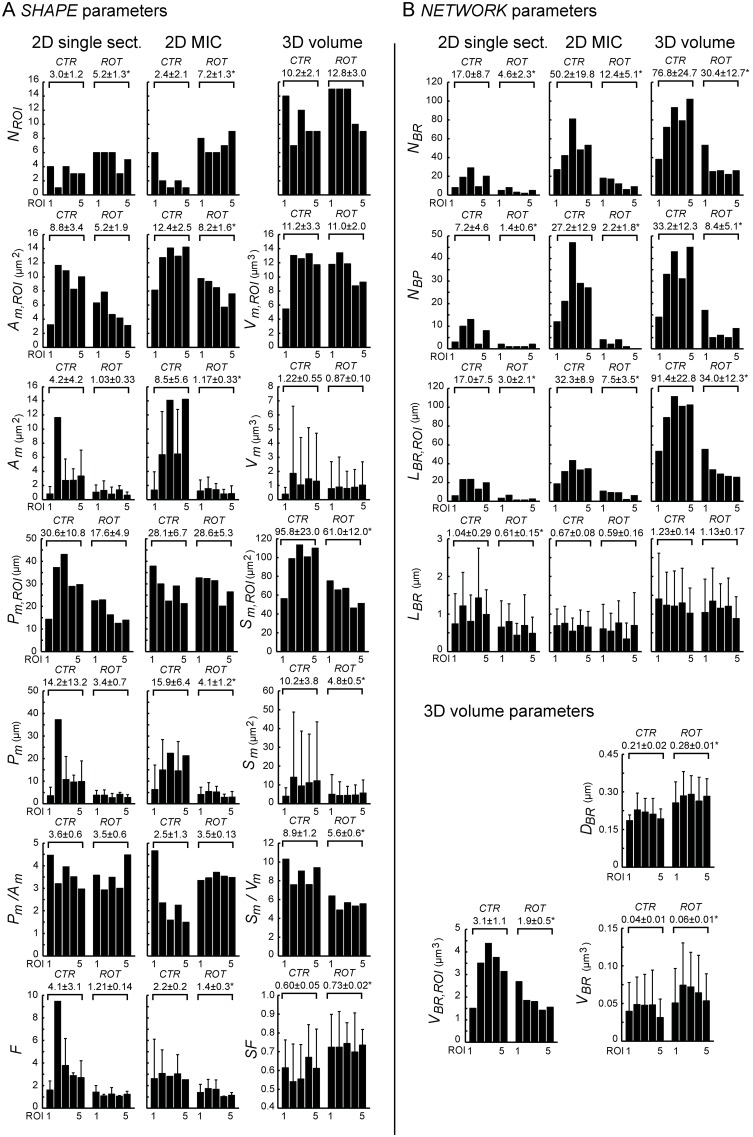
Shape and network analysis of normal *vs.* stressed HUVEC mitochondria. The images from the study in **Fig. 6** were analyzed with respect to 2/3D “*SHAPE”* (**A**) and “*NETWORK”* parameters (**B**). The positions of the different ROIs are shown in **Fig. 6B**. The data resulting from 2D analysis of the highest intensity frame (“2D single sect.”) and the z-stack MIC (“2D MIC”) where compared with the 3D data (“3D volume”). The descriptor variables are explained in **Table 1**. *p<0.05 compared to CTR, Student’s t-test, two-tailed.

2D shape analysis (MIC images) demonstrated a 3-fold increase in *N_ROI_* in the stressed cell compared to the normal cell, whereas 3D analysis did not suggest a significant effect (**Fig. 7A**). Furthermore, 2D shape analysis (MIC images) demonstrated a significant decrease in *A_m,ROI_* and *A_m_* in the stressed cell. However, 3D analysis revealed no change in the corresponding volumetric parameters (*i.e. V_m,ROI_* and *V_m_*) although the mean value of *V_m_* displayed a lower standard deviation (SD). The latter is compatible with mitochondria having a more uniform size distribution in stressed cells, supported by the smaller mean and SD value of *S_m_*. A similar reduction in mean value was also observed for *P_m_* (2D MIC image). Although the *P_m,ROI_* calculated from the 2D MIC image was unchanged in stressed cells its corresponding 3D parameter (*S_m,ROI_*) was reduced. Calculation of the *P_m_/A_m_* and *S_m_/V_m_* ratios supports the visual observation that mitochondrial shape is changed in the stressed situation. Similarly, *F* and *SF* both indicate that mitochondria were more circular (2D) or spherical (3D) in the stressed cell.

The 2D and 3D quantification of mitochondrial network properties discriminated well between the two mitochondrial phenotypes in normal and stressed cells (**Fig. 7B**). In this sense, *N_BR_*, *N_BP_* and *L_BR,ROI_* were all significantly reduced in the stressed cell. However, the total branch length per ROI (*L_BR,ROI_*) was only about one third in 2D MIC analysis compared with the 3D analysis, in both cell types. The difference was significantly less for the two branching descriptors (*N_BR_*, and *N_BP_*). The mean value of *L_BR_* (2D MIC image and 3D) was not changed in the stressed cells. Although the total mitochondrial branch volume (*V_BR,ROI_*) was reduced in the stressed cell, the “thickness” (*D_BR_*) and size (*V_BR_*) of the mitochondrial branches were increased. In summary, both the 2D and the 3D analysis clearly demonstrated significant reduction in the number, length and branching points of mitochondrial filaments in the stressed cell compared to the normal cell.

**Figure 8:**
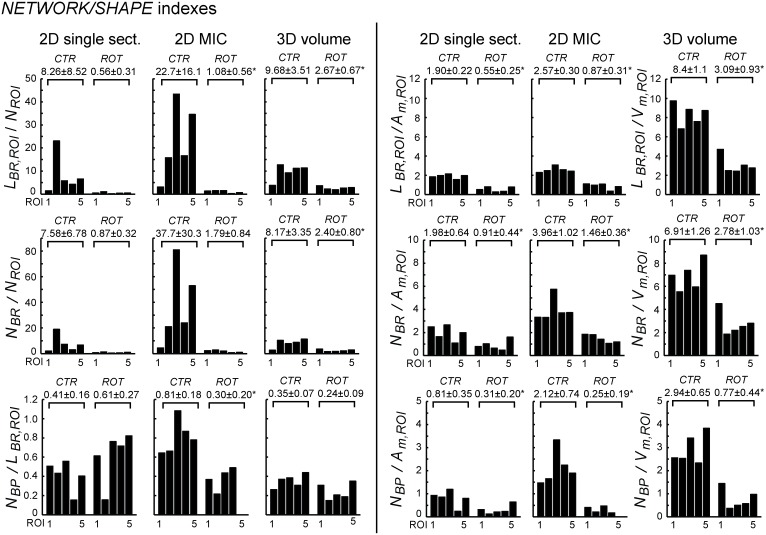
Integrative network/shape analysis of normal *vs.* stressed HUVEC mitochondria. Based on the analysis performed in **Fig. 6** and **Fig. 7**, integrative “*NETWORK/SHAPE”* indexes were calculated as described in **Table 1**.

In order to maximize the amount of information, we integrated the data from mitochondrial shape and network analysis by calculating the following derived parameters (**Table 1**): (**i**) the branch length per mitochondrion (equaling *L_BR,ROI_/N_ROI_*; calculation possible in 2D and 3D), (**ii**) the branch length per biomass (equaling *L_BR,ROI_/A_m,ROI_* in 2D and *L_BR,ROI_/V_m,ROI_* in 3D), (**iii**) the number of branches per mitochondrion (equaling *N_BR_/N_ROI_*), (**iv**) number of branches per biomass (equaling *N_BR_/A_m,ROI_* in 2D and *N_BR_/V_m,ROI_* in 3D), (**v**) branching point frequency relative to branch length (equaling *N_BP_/L_BR,ROI_*), (**vi**) branching point frequency relative to biomass (equaling *N_BP_/A_m,ROI_* in 2D and *N_BP_/V_m,ROI_* in 3D) (**Fig. 8**).

The overall effects of stress on the derived parameters were similar for 2D and 3D analyses. The ratios calculated relative to biomass (*A_m,ROI_* in 2D and *V_m,ROI_* in 3D) were found to be particularly consistent and of relatively low variability both in 2D and 3D analysis. In the 3D case, this was also true for the other indexes. Taken together, calculation of ratios that combine parameters of mitochondrial shape and network properties revealed that (the) mitochondria (l) (network) consisted of fewer, shorter and less branched filaments in the stressed cell. This supports the conclusion that mitochondrial morphology changes from a reticular state to circular/spherical organelles in the stressed cell.

## Discussion

Image-based 2D analysis of mitochondrial objects has proven to be a valuable strategy in flat cells such as primary human skin fibroblasts with an axial dimension ≤3 µm [6], [20], . In this study, we evaluated methods of 2D *vs.* 3D analysis in relatively thick cells (HUVECs), and compared the quantitative outcome. Furthermore, we established and validated a strategy for integrated quantification of mitochondrial shape and network properties in adherent cells with a non-flat morphology; for applications both in 2D and 3D analysis. The method combines well-established image processing operations to allow segmentation and detailed analysis of mitochondrial objects. Results from intact cells demonstrated that this approach provided new information about mitochondrial morphology and topology.

### Performance of deconvolution and FFT filtering in 2D and 3D analysis

The performance of deconvolution and/or FFT filtering was evaluated in prototypic non-flat cells (HUVECs) expressing mitoGFP targeted to the mitochondrial matrix of cells. Given the time required for acquisition, cells were fixed to prevent mitochondrial movement. We observed that 3D deconvolution significantly improved the segmentation of mitochondrial objects in the z-stack, and thereby the quality of the shape and network analysis. The characteristics of the z-stack fluorescence intensity histogram were less affected by deconvolution when compared to the spatial filtering procedure. However, subsequent 2D quantification of mitochondrial parameters in the individual z-stack sections yielded similar results for deconvolved and spatially processed images. In the 3D case, deconvolution allowed a more robust mitochondrial segmentation and analysis. These results demonstrate that image optimization by deconvolution constitutes a valid alternative to the 2D spatial filtering strategy prior to 2D analysis. In contrast, our results revealed that 3D deconvolution is the preferred strategy for z-stack processing prior to 3D analysis. FFT filtering has been successfully applied in other studies that did not use deconvolution [28]. However, we did not include FFT filtering in our image processing scheme since it did not (further) improve the analysis.

### Integrative analysis of filamentous *vs.* non-filamentous HUVEC mitochondria

The developed 3D protocol was benchmarked by comparing mitochondrial shape and network parameters between z-stacks of filamentous (“Normal”) and non-filamentous (“Stressed”) HUVEC mitochondria. We also tested several semi-3D strategies by collapsing multiple z-stack sections into a representative 2D image. Clearly, such an approach is technically less demanding and time-consuming than full-scale z-stack 3D analysis. The difference between the MIC projection of all the sections compared with the two 3-sections projections (MIC and Avg) was relatively small. It was, however, evident that some features were lost or rendered in the projected images compared to the 3D analysis. Obviously, projecting an entire z-stack into a single 2D image can lead to excessive merging/shielding of objects, which is undesirable. Analysis of the single highest intensity z-stack section was less reliable compared to the MIC image when examining mitochondrial morphology in normal and stressed cells. This means that although z-stack projection approaches may be employed to study certain aspects of mitochondrial morphology, this analysis should preferably be supported by complementary methods [6]. In this study, we focused on comparing data from the 2D MIC image with results from 3D volume analysis. Although the quantified data were relatively consistent between the different ROIs of the same cell, some variation was observed, such as for ROI 1 of the normal cell (**Fig. 7**). For this particular ROI, the deviation may be explained by its peripheral position near the border of the mitochondrial reticulum, whereas the other ROIs included more central parts and yielded more similar quantitative data (**Fig. 6B**). When applicable, the internal variation within each ROI was also evaluated.

Compatible with visual observations, both 2D and 3D analysis indicated a shift from filamentous network morphology to circular (in 2D) and spherical (in 3D) organelles. This effect was particularly clear from the network analysis, since both 2D and 3D descriptors indicated a reduction in the number of mitochondrial branches (*N_BR_*), mitochondrial branching points (*N_BP_*), and total mitochondrial branch length (*L_BR,ROI_*). 2D mitochondrial shape analysis suggested that stressed cells contained a higher number (*N_ROI_*) of smaller (*A_m_*) and more circular (*F*) mitochondria, accompanied with a loss in mitochondrial biomass (*A_m,ROI_*). This indicates that mitochondrial fragmentation and removal occurred in the stressed cells. In contrast, 3D mitochondrial shape analysis revealed a morphological change towards more spherical organelles (*SF*) without alterations in volume of individual mitochondria (*V_m_*) and total mitochondrial biomass (*V_m,ROI_*). This suggests that mitochondria are swollen but not fragmented in the stressed cell, supported by the increase in mitochondrial branch volume (*V_BR_*).

The decrease in mitochondrial number (*N_ROI_*) suggested by the 2D analysis, in contrast to 3D volume analysis, was most likely due to axial branch/object crossing between individual optical z-sections leading to artifactual object merging in the 2D MIC projection (**Fig. 7A**). This phenomenon will predominantly affect elongated filamentous mitochondria when the 2D projection (*i.e.* MIC) is created from the z-stack, thereby leading to an erroneous apparent reduction in *N_ROI_*. Another reason why *A_m_* (2D), but not *V_m_* (3D), was affected, may relate to the fact that the stressed cell was “rounding” up (*i.e.* became “thicker”). The latter will alter the axial mitochondrial distribution, and thereby increase the merging/shielding of organelles in the collapsed 2D MIC image. Our observation that *V_BR_* and *V_BR,ROI_* were differently affected is likely explained by the fact that fewer mitochondrial branches were detected and measured in the stressed cell, which reduces total branch volume (*V_BR,ROI_*) but not necessarily branch mean value (*V_BR_*). The same reason is valid for explaining the reduction in total branch length (*L_BR,ROI_*), but not the branch mean value (*L_BR_*).

By comparing two characteristic mitochondrial phenotypes (i.e. filamentous *vs.* non-filamentous mitochondria) we were able to judge in detail the implications of the different processing steps for the mitochondrial quantification. Of note, the effects of ROT on mitochondrial morphology seem to depend on cell type, ROT concentration and exposure time [21], [22]. Hence, additional systematic studies are required to characterize in detail the biological effects of ROT in HUVECs, including statistical analysis addressing heterogeneity in the cultures.

### Summary and conclusions

This study demonstrates that both 2D and 3D analyses can be employed to discriminate and characterize changes in mitochondrial shape and network properties. In this sense, both types of analysis supported a change from a filamentous to a non-filamentous mitochondrial morphology in normal *vs.* stressed HUVECs, in agreement with visual observations. However, 2D and 3D analysis led to contradictory conclusions regarding mitochondrial fragmentation. The results confirmed that 3D analysis is the preferred method in studies of mitochondria in non-flat adhering cells. Integrative analysis of object shape and network properties was found to provide new insight into important aspects of mitochondrial dynamics. This strategy may potentially be adapted to other biological contexts and imaging modalities in order to gain new knowledge about mitochondrial (patho) physiology.

## Supporting Information

Video S1 **3D volume model of endothelial mitochondria.** The video shows a 3D volume representation of mitochondrial fluorescence in the z-stack analyzed in Figure 2, 3 and 4, which was acquired from a mitoGFP expressing HUVEC by confocal microscopy. The z-stack was processed by 3D blind deconvolution and a contrast stretch (as described in the article).
(WMV)

Video S2 **3D surface (shape) model of endothelial mitochondria.** The video shows a 3D model of the mitochondrial surface in the same z-stack as **Video S1** (*i.e.* a mitoGFP expressing HUVEC). An intensity threshold value (surface value) was applied to assign the surface.
(WMV)

Video S3 **3D network model of endothelial mitochondria.** The video shows a 3D model of the mitochondrial network in the same z-stack as **Video S1** (*i.e.* a mitoGFP expressing HUVEC). The mitochondrial filaments were skeletonized and vectorized, and mitochondrial branches (green branches), branching points (purple dots, 3 branches; red dots, 4 branches) and endpoints (green dots) were identified.
(WMV)
